# EEG neural oscillatory dynamics reveal semantic and response conflict at difference levels of conflict awareness

**DOI:** 10.1038/srep12008

**Published:** 2015-07-14

**Authors:** Jun Jiang, Qinglin Zhang, Simon Van Gaal

**Affiliations:** 1 Department of Basic Psychology, School of Psychology, Third Military Medical University, Chongqing, China; 2 Key laboratory of cognition and personality (Ministry of Education), and Faculty of psychology, Southwest University, Chongqing, China; 3 University of Amsterdam, Department of Psychology, the Netherlands; 4 Donders Institute for Brain, Cognition and Behavior, the Netherlands

## Abstract

Although previous work has shown that conflict can be detected in the absence of awareness, it is unknown how different sources of conflict (i.e., semantic, response) are processed in the human brain and whether these processes are differently modulated by conflict awareness. To explore this issue, we extracted oscillatory power dynamics from electroencephalographic (EEG) data recorded while human participants performed a modified version of the Stroop task. Crucially, in this task conflict awareness was manipulated by masking a conflict-inducing color word preceding a color patch target. We isolated semantic from response conflict by introducing four color words/patches, of which two were matched to the same response. We observed that both semantic as well as response conflict were associated with mid-frontal theta-band and parietal alpha-band power modulations, irrespective of the level of conflict awareness (high vs. low), although awareness of conflict increased these conflict-related power dynamics. These results show that both semantic and response conflict can be processed in the human brain and suggest that the neural oscillatory mechanisms in EEG reflect mainly “domain general” conflict processing mechanisms, instead of conflict source specific effects.

Accumulating evidence shows that many cognitive and perceptual functions can be influenced by unconscious information^1^,^2^,^3^. However, at the same time, research has shown that awareness of information strongly modulates the extent to which participants can use this information for decision-making and complex behavior^4^,^5^,^6^,^7^. In this paper, we will focus on the role of awareness in a cognitive control process called “conflict detection”. Overall, conflict can arise from several sources, including sensory and motor representations and resolving conflict to optimize behavior is a core cognitive control function^8^,^9^,^10^. By combining a modified version of the Stroop task with oscillatory power analyses on electroencephalographic (EEG) data we aimed to test two specific questions: 1) To what degree is conflict control implemented by domain general or domain specific neural (oscillatory) mechanisms, and 2) to what extent are neural oscillatory processes related to the different forms of conflict processing (sensory and motor) modulated by conflict awareness.

Using various versions of the masked priming tasks, previous studies have revealed that prime-induced conflict can affect the speed and accuracy of behavioral responses and neural modulations related to conflict can be detected in the absence of awareness^3^,^11^,^12^,^13^,^14^,^15^,^16^. In a typical conflict experiment, participants are required to perform a speeded two-choice response to a target stimulus that can be preceded by a so-called prime stimulus. For example, in a Stroop priming task, a modified version of the classic Stroop task, the prime can be a color word printed in black (“red”/”blue”) followed by a color patch that is the target^16^,^17^,^18^. Typically, one color is mapped onto the right hand and the other color to the left hand. Prime awareness can be modulated for example by masking. In behavior, in these and similar experiments, participants respond faster and more accurate to congruent prime-target pairs compared to incongruent pairs, referred to as (unconscious) priming^3^,^12^,^19^,^20^. Recent electroencephalographic (EEG) studies have shown that even unconscious conflict elicits several ERP components, for example the conflict-related mid-frontal N2^19^ and the fronto-parietal N450^16^. Further, neuroimaging studies have also shown that unconscious conflict can activate the “conflict monitoring system” in the medial frontal cortex, including the anterior cingulate cortex (ACC)^21^,^22^,^23^, the dorsal lateral prefrontal cortex (DLPFC) and the parietal cortex^21^,^24^. Although unconscious conflict detection seems to be a well-established effect by now, there is less consensus about how such conflict priming actually works and to what extent awareness of conflict changes the processing of conflict information in the human brain.

After considerable research and debate, it seems that both sensory/semantic conflict (i.e., the semantic incongruency between the word and the color patch in the previous example) and response conflict (the fact that the prime and target are mapped to different response hands) contribute to (unconscious) priming effects^12^,^25^,^26^. However, at present, it is disputed to what extent cognitive control over different sources of conflict (sensory/motor) is implemented by shared (domain general) or distinct (domain specific) neural processes, or whether it is a mix of both. Using conflict dissociation procedures (explained below) with conscious stimuli only, previous EEG and functional magnetic resonance imaging (fMRI) studies have revealed a mixed picture of the neural mechanism underlying the processing of semantic and response conflict. To illustrate, van Veen and colleagues^8^ found that the fronto-central N2 ERP component was enhanced by response conflict only and Augustinova and colleagues^27^ found that the frontal N450 was only sensitive to semantic conflict, suggesting a dissociation between the neural mechanisms underlying both sources of conflict. In an fMRI study, van Veen and colleagues^10^ revealed that semantic conflict and response conflict both activate the ACC, the DLPFC and the posterior parietal cortex (PPC), however non-overlapping and distinct subsystems thereof. Soutschek and colleagues^28^ directly examined the neural mechanisms of stimulus conflict and response conflict using transcranial magnetic stimulation (TMS). They observed that stimulating the posterior intraparietal sulcus/inferior parietal lobule modulated the magnitude of semantic conflict, whereas stimulating the pre-SMA modulated the magnitude of response conflict. These studies seem to suggest that there are conflict-specific control networks for semantic and response conflict. However, as already mentioned, evidence is mixed. Some EEG studies have shown that the N2^29^ and N450^30^ ERP components are both sensitive to semantic and response conflict. EEG time-frequency decomposition also showed that the power of medial frontal theta (4–8 Hz) was sensitive to both semantic and response conflict^31^. In a recent EEG study, Wang and colleagues^32^ have found that both stimulus conflict and response conflict enhanced medial frontal theta-band and alpha band power (response conflict only triggered medial frontal beta-band modulations). A recent fMRI study using neural pattern classifiers^33^ has shown that the human control system might actually be a hybrid neural architecture that includes both domain specific and domain general components. Based on these and other studies, it is as yet undecided if and to what extent conflict control networks of the medial/lateral frontal cortex and parietal cortex are different for processing semantic and response conflict^32^,^33^.

In this paper we aim to further explore how both sources of conflict are processed in the human brain using EEG and whether those sources might differ for conflict at different levels of awareness. To do so, we dissociated semantic and response conflict in a masked Stroop priming task and manipulated the level of awareness of the color-word by masking^16^. Contrary to a typical color-word Stroop task that has only two colors and two response options, we used a version similar to De Houwer^9^, which has four colored words, but only two response options (Fig. 1A). Thereby, there are three relevant conditions in this experiment: the congruent condition (CO: the word meaning is the same as the printed ink color and are mapped to same response button), the semantically incongruent condition (SI: word meaning and printed color are different, but mapped to the same response button) and the response incongruent condition (RI: meaning and printed color are different and mapped to different response buttons). Therefore, the comparison between SI and CO reflects pure semantic conflict (different colors but mapped to the same response), whereas the contrast between SI-RI reflects response conflict only (both have semantic conflict)^8^,^9^,^10^,^27^,^30^,^34^ (see Fig. 1B). By manipulating the masking strength of the color-word we created two quantitatively different awareness levels, which we refer to as low visible and high visible conflict trials. However, recently, it has been demonstrated that most of the conflict-related EEG signal is non-phase-locked^35^,^36^ and therefore is averaged out while calculating ERPs. Because spectral analyses are more sensitive to conflict than ERP analyses and might reveal subtle modulations (for example in low visible trials) that cannot be revealed by ERPs we decided to focus on spectral analyses only in the present manuscript.

## Methods

### Participants

Twenty-two undergraduate students (10 females) aged between 19 and 24 (M = 21.00, SD = 1.54) were recruited from campus intranet of the Southwest University of China. They participated for monetary compensation. All participants were right-handed, had normal or corrected-to-normal vision and normal color vision, and had no history of head injury. The local ethics committee of Southwest University approved this study. In accordance with the approved guidelines, written informed consent was obtained from all participants after the explanation of the experimental protocol.

### Apparatus and stimuli

**Figure 1:**
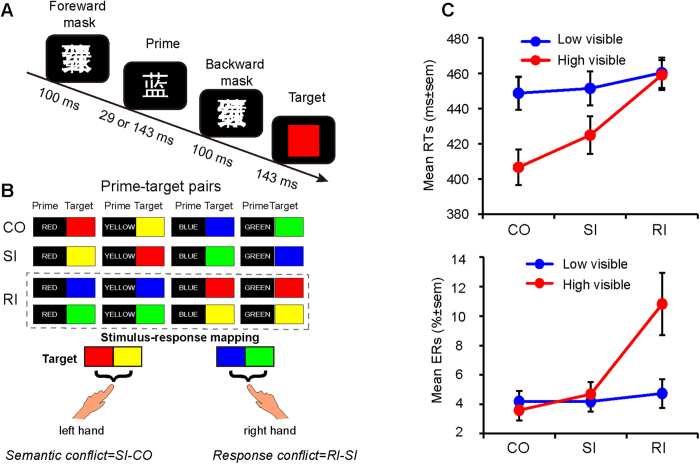
Experimental design and behavioral results. (**A**) Schematic representation of the experimental task and the stimuli. Primes could be congruent (or incongruent) with the semantic meaning or response-mapping of the target (50% trials are CO, 25% trials are SI, 25% trials are RI). Primes could be presented briefly (29 ms, strongly masked primes) or longer (143 ms, weakly masked primes). (**B**) Examples of trials. (**C**) Mean reaction times (RTs, upper panel) and mean error rates (ERs, lower panel) for CO, SI and RI trials under different levels of conflict awareness. Error bars reflect the standard error of the mean. CO = Congruent; SI = Semantic Incongruent; RI = Response Incongruent. Note: In the actual experiment the background of the computer screen was gray, not black.

All stimuli were presented against a gray (RGB: 128, 128, 128) background at the center of a 17-inch Lenovo CRT monitor (frequency 70 Hz, resolution 1024 × 768) with the E-prime 1.1 software package (Psychology Software Tools, Pittsburgh, PA). Participants were seated ~60 cm from the computer screen and performed a masked Stroop priming task, in which the primes were four Chinese color words (“red”, “yellow”, ”blue”, ”green”) printed in white that appeared in Song font (font size 36), extending a visual angle of 0.96° × 1.05°. Masks were constructed by first overlapping the four color words, then one of them was randomly selected and inverted (visual angle: 1.06° × 1.16°). The targets were four color patches (“red”, “yellow”, ”blue”, ”green”) with the same size as the masks (Fig. 1A,B).

### Design and behavioral procedures

A forward mask was first presented for 100 ms, followed by a prime that was presented for 29 ms (low visibility) or 143 ms (high visibility). Next, a backward mask (identical to forward mask) appeared for 100 ms, followed by the target stimulus (presented for 143 ms). The post-target interval was jittered between 1200–1500 ms (see Fig. 1A). In half of the trials the prime was strongly masked, and in the other half it was weakly masked. Participants were instructed to ignore the prime and to respond as quickly and accurately as possible to the color target by pressing a key on the standard QWERTY keyboard. Specifically, half of the participants were asked to press “F” with the left index finger if the color of target was either red or yellow, and if the color of target was blue or green they were instructed to press “J” with the right index finger. The stimulus-response mapping was reversed in the other half of the participants. According to the color and response mapping, the trials can be categorized into congruent (CO, the mean of prime word same as the target and the evoked response also same), semantic incongruent (SI, the mean of the prime was not same as the target color, but the response evoked by the prime and target were same), response incongruent (RI, the mean of the prime was not same as the target color, and the response evoked by the prime and target were also different) (Fig. 1B). The proportion of the three conditions is CO (50%), SI (25%), RI (25%)^8^,^10^. High and low visible trials were mixed within blocks. Therefore, there were two factors: Visibility (high, low) and Congruency (CO, SI, RI) of interest for our analyses.

Before the actual experiment, participants performed one practice block of 24 trials with performance feedback (mean RT and percentage correct) after each trial. Thereafter, they performed 8 experimental blocks of 192 trials each (1536 trials in total). Then, at the end of the experiment, the participants performed a two-alternative forced-choice (2AFC) discrimination task (256 trials, the ratio of high and low visibility trials was 1:1) to test the visibility of the primes. In this block, participants were informed that in all trials actually there was a color word presented between the forward and backward masks. Participants were asked to report the meaning of prime word. The timing of the stimuli and the response rule were same as the actual experiment (but now dedicated to the prime word instead of the target). For example, if the meaning of prime word is red or yellow, they should press ‘F’ on the keyboard with left index finger, while if the meaning of prime is green or blue, they should press ‘J’ with right index finger. All trials were presented randomly.

### Behavioral data analysis

Incorrect, missed and correct trials with reaction time <200 or >1000 ms were excluded from RTs analyses (5.0%). Reaction times (RTs) and error rates (ERs) were separately submitted to two repeated measures ANOVAs with Visibility (high, low) and Congruency (CO, SI, RI) as within-subject factors. A one sample t-test on the discrimination accuracy or d’ was used to analyze prime visibility. A two-tailed significance level of 0.05 was used for all behavioral tests.

### EEG measurements and preprocessing

Participants were seated in a dimly lit and electrically shielded room and were asked to avoid eye blinks and movements when the stimulus was presented. EEG activity was recorded from 64 scalp sites using tin electrodes mounted in an elastic cap (Brain Products, Munich, Germany) with the references on FCz and a ground electrode on AFz. The vertical electro-oculogram (EOG) was recorded from an electrode below the right eye. The horizontal EOG was collected from an electrode located at the outer canthus of the right eye. EEG and EOG signals were filtered using a 0.01–100 Hz band-pass and continuously sampled at 500 Hz. The impedance of all electrodes was kept below 5 kΩ.

The preprocessing was conducted using custom-made MATLAB (R2013a, The MathWorks, Inc.) scripts based on an open source software EEGLAB^37^. Firstly, continuous EEG data were offline digitally filtered with band-pass between 0.5–90 Hz and re-referenced to the average of the activity recorded at the left and right mastoids, and then segmented from −1.5 s to 2 s relative to target onset. The epochs corresponding to the behavioral exclusion criteria and epochs deviating more than 5 SD from the mean probability distribution were excluded^15^. Finally, independent components (ICs) were computed using the runica function of EEGLAB to isolate artifacts contained in the epoched EEG data. ICs representing eye blinks, eye movements, muscle artifacts, or other types of noise were removed from the EEG signal (mean number of removed IC across subjects = 3.55).

### Time frequency decomposition

The clean EEG data were first current-source-density (CSD) transformed, which is a spatial filter that minimizes volume-conducted effects and increases topographical selectivity by effectively removing spatially broad signals. The method has been validated in previous EEG studies^38^,^39^. Single-trial EEG data of each condition were then decomposed into their time-frequency representations (TFRs) from 1 Hz to 40 Hz in 40 logarithmically spaced steps using the custom-made Matlab scripts, which first multiply the power spectrum of the EEG (obtained from the fast Fourier transform) by the power spectrum of complex Morlet wavelets (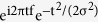, where t is time, f is frequency, and σ defines the width of each frequency band, which was set as 3–7 logarithmically spaced cycles to trade-off temporal and frequency resolution), and then taking the inverse fast Fourier transform. From the resulting complex signal, an estimate of frequency band-specific power at each time point was defined as the squared magnitude of the result of the convolution Z (real[z(t)]^2^ + imag[z(t)]^2^). The TFRs of each subject of each condition were first averaged and then was transformed using a decibel (dB) scale and normalized using the common baseline (averaged across all conditions, −550 ms to −350 ms before the target onset) activity for each estimated frequency according to the equation: dB power = 10*log10 (power/baseline)^38^. Conversion to a dB scale ensures the data across conditions can be statistically comparable in the same scale.

### Time frequency power analysis

**Figure 2:**
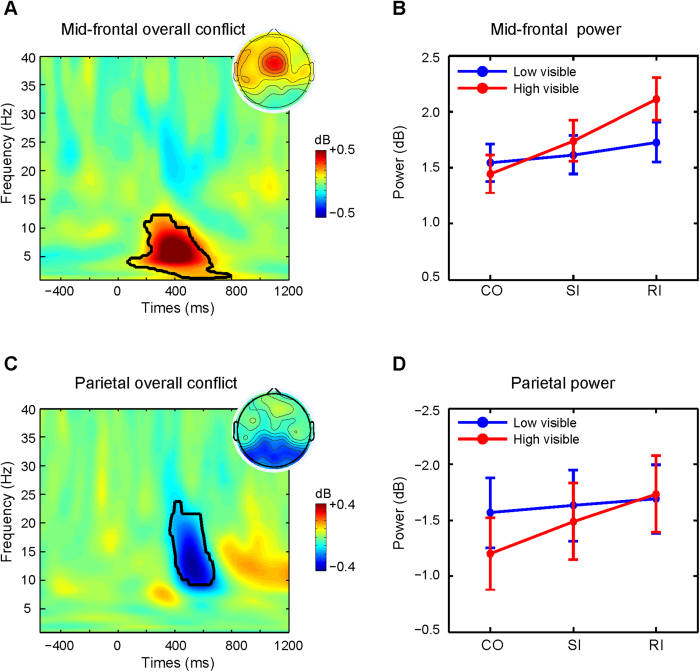
Semantic and response conflict effects at the mid-frontal and parietal region of interest. (**A**) Time-frequency power dynamics of overall conflict ((RI + SI)-CO) averaged across all prime visibility conditions at the mid-frontal ROI. Time 0 is the onset of the target. Black lines encircle regions of significance at p < 0.001, corrected for multiple comparisons using cluster-based statistics (see Methods). The averaged theta-band power data in the enclosed significant area were used for the topographical plot shown in upper right corner. (**B**) Line plot for the power of the significant time-frequency region of interest (all pixels encircled in A) at mid-frontal ROI separated by visibility and congruency. (**C**) Time-frequency power dynamics of overall conflict ((RI + SI)-CO) averaged across all prime visibility conditions at the parietal ROI. Time 0 is the onset of the target. Black lines encircle regions of significance at p < 0.001, corrected for multiple comparisons using cluster-based statistics. The averaged alpha-band power data in the enclosed significant area were used for the topographical plot shown in upper right corner. (**D**) Line plot for the power of the significant time-frequency region of interest (all pixels encircled in **C**) at the mid-frontal ROI separated by visibility and congruency. The error bars represent the mean standard error (SEM). CO = Congruent; SI = Semantic Incongruent; RI = Response Incongruent.

**Figure 3:**
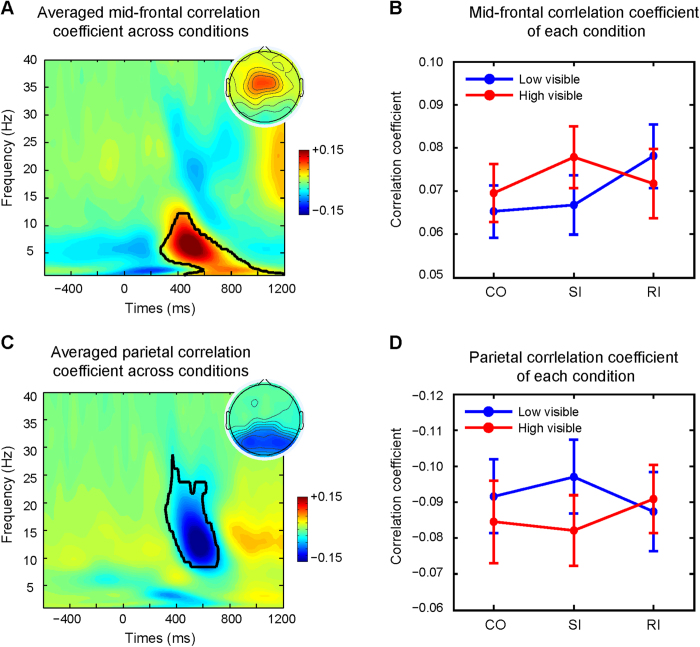
Time-frequency plots of the correlation between single-trial power and RT across all conditions at the mid-frontal ROI (top) and the parietal ROI (bottom). (**A**) Time-frequency correlation coefficient plot averaged across all conditions at mid-frontal ROI. Time 0 is the onset of the target. Black lines encircle regions of significance at p < 0.001, corrected for multiple comparisons using cluster-based statistics. The averaged theta-band RT correlation coefficient in the enclosed significant area was used for the topographical plots shown in the upper right corner. (**B**) Line plot for the correlation coefficient of the significant time-frequency region of interest (all pixels encircled in A) at mid-frontal ROI separated by visibility and congruency. (**C**) Time-frequency correlation coefficient plot averaged across all conditions at parietal ROI. Time 0 is the onset of the target. Black lines encircle regions of significance at p < 0.001, corrected for multiple comparisons using cluster-based statistics. The averaged alpha-band correlation coefficient in the enclosed significant area was used for the topographical plot shown in upper right corner. (**D**) Line plot for the correlation coefficient of the significant time-frequency region of interest (all pixels encircled in **C**) at mid-frontal electrodes separated by visibility and congruency. The error bars represent the mean standard error (SEM). CO = Congruent; SI = Semantic Incongruent; RI = Response Incongruent.

Based on previous studies, we created two region of interests (ROIs): a “mid-frontal ROI” (Fz, FCz, FC1, FC2, Cz) where theta-band conflict effects tend to peak and a “parietal ROI” (p7, p5, p3, p1, pz, p2, p4, p6, p8, po7, po3, poz, po4, po8) where alpha/beta-band conflict effects tend to peak^28^,^29^,^30^,^40^,^41^,^42^,^43^. Statistics on the overall Stroop priming effect ((SI + RI)-CO) were performed by t-tests. We corrected for multiple comparisons by performing cluster-based permutation tests^44^,^45^, in which the assignment of a condition to each data point was randomly shuffled, and statistics were re-computed. After thresholding each permutation map (p = 0.001), the number of pixels in the largest supra-threshold cluster was stored. This procedure was repeated 2000 times, generating a distribution of maximum cluster sizes under the null hypothesis. Any clusters in the real data that were at least as large as the 95% of the distribution of null hypothesis cluster sizes were considered statistically significant. Based on statistical results shown in Fig. 2A and 3A, the data enclosed by the contour line (the significant cluster) in each condition of each subject were first extracted and averaged, and then submitted to repeated ANOVAs with Visibility (high, low) and Congruency (CO, SI, RI) as within-subject variables.

### Trial-to-trial correlations with RT

To examine whether time frequency power dynamics were correlated with RTs in the different conditions, we calculated Spearman rank correlations for single subjects (across trial correlations between single trial power and single trial RT for each condition). This produces a time-frequency map of correlation coefficients for each subject and each condition. These single subject maps were averaged across subjects and conditions for each time-frequency point. The obtained correlation coefficients were Fisher-Z transformed before we performed group-level analyses^45^. Correlation coefficients at each time-frequency pixel were tested against zero at the group level and then corrected for multiple comparisons using cluster-based permutation tests (same as the above mentioned procedure).

## Results

### Behavioral results

A repeated measures ANOVA on mean RTs and error rates (ERs) revealed a main effect of Visibility (RT:F_1,21_ = 255.87, p < 0.001; ER:F_1,21_ = 21.27, p < 0.001), meaning that participants responded slower (23 ms) and committed more errors in high visible (RT:M = 430 ms, SE = 9.10; ER:M = 4.36%, SE = 0.72%) than in low visible (RT:M = 453 ms, SE = 8.91; ER:M = 6.36%, SE = 0.98%) trials. Also the main effect of Congruency (RT:F_2,42_ = 54.16, p < 0.001; ER:F_2,42_ = 9.05, p = 0.001) was significant. Follow-up analyses revealed that both semantic conflict (SI-CO; RT:M = 11 ms, t_21_ = 6.60, p < 0.001; ER:M = 0.55%, SE = 0.27%; t_21_ = 2.04, p < 0.05) and response conflict (RI-SI; RT:M = 21 ms, t_21_ = 5.89, p < 0.001; ER:M = 3.34%, SE = 1.23%; t = 2.79, p < 0.01) were significant across visibility conditions, indicating the occurrence of both types of conflict. As expected, prime visibility modulated both semantic and response conflict effects indexed by a significant two-way interaction (RT:F_2,42_ = 46.21, p < 0.001; ER:F_2,42_ = 15.79, p < 0.001). Further analyses on the interaction of RTs showed that response conflict was significant regardless of prime visibility (low visible: M = 9 ms, t_21_ = 3.31, p = 0.003; high visible: RT:M = 34 ms, t_21_ = 6.43, p < 0.001), whereas semantic conflict was only significant in high (M = 18 ms, t_21_ = 6.85, p < 0.001), but not in low (M = 3 ms, SE = 1.97; t_21_ = 1.41, p = 0.173) visible trials (Fig. 1B upper panel). Further analysis on the interaction of ERs revealed that both semantic conflict (M = 1.09%, SE = 0.39%; t_21_ = 2.77, p = 0.012) and response conflict (M = 6.14%, SE = 1.94%; t_21_ = 3.16, p = 0.005) were significant in high visible trials, but none was significant (ts < 1) in low visible trials (Fig. 1B lower panel). Direct comparisons between semantic conflict and response conflict for each visibility level showed that response conflict was larger than semantic conflict in high visible trials (RT:t_21_ = 2.68, p = 0.014; ER:t_21_ = 2.43, p = 0.024), but not in low visible trials (RT:t_21_ = 1.46, p = 0.159; ER:t < 1). Note that the pattern of results of the high visibility condition for both RT and ER directly replicate the behavioral data pattern observed by van Veen and Carter^8^ (their Fig. 1) using a modified version of the flanker task.

### Time-frequency results

As expected, the analyses on overall conflict (Stroop priming effect irrespective of visibility: (RI + SI) - CO) revealed two significant spatial-time-frequency clusters. The first cluster was centered at fronto-central electrodes (“mid-frontal ROI”) and revealed typical conflict-related theta-band power modulations (frequency range: 1.3–11.7 Hz, time range: 70–790 ms, peak power = 0.62 dB, peak frequency = 5.5 Hz, peak time = 410 ms, see Fig. 2A). The second cluster was centered at occipito-parietal electrodes (“parietal ROI”) and revealed an alpha/beta-band reduction, in high alpha (frequency range: about 8.8–22.7 Hz, time range: about 370–680 ms, peak power = −0.38 dB, peak frequency = 12.9 Hz, peak time = 520 ms, see Fig. 2C). Note that our analysis procedure of first selecting Time-Frequency ROI’s (theta and alpha) and spatial ROI’s (mid-frontal and parietal) based on the general contrast of “conflict vs no conflict” allows us to directly compare the six experimental conditions in ANOVA’s (Conflict (3) by Visibility (2), see below). However, to be sure that there were no strong topographical and/or spectral differences between response conflict and semantic conflict we have also performed the conflict-specific contrasts: RI-SI and SI-CO (see supplementary Figs S1 and S2). These contrasts revealed that semantic conflict and response conflict enhanced mid-frontal theta and parietal alpha in a highly similar manner.

Next, we further zoomed in on these separate effects to examine differences in the source of conflict and awareness-related differences. A repeated measures ANOVA on mid-frontal theta power yielded a main effect of Visibility (F_1,21_ = 14.06, p < 0.001) and Congruency (F_2,42_ = 64.07, p < 0.001), as well as the two-way interaction (F_2,42_ = 21.73, p < 0.001). Further analyses indicated that, relative to the baseline, high visible trials (M = 1.76 dB, SE = 0.18) evoked more theta power than low visible trials (M = 1.63 dB, SE = 0.17). Further analyses on the interaction between Congruency and Visibility showed that response conflict (M = 0.11 dB, SE = 0.05, t_21_ = 2.27, p = 0.034), but not semantic conflict (M = 0.07 dB, SE = 0.04, t_21_ = 1.79, p = 0.09), was significant in low visible trials, whereas both semantic (M = 0.30 dB, SE = 0.04, t_21_ = 6.97, p < 0.001) and response conflict (M = 0.37 dB, SE = 0.06, t_21_ = 6.10, p < 0.001) were significant in the high visible trials (Fig. 2B). Planned paired t-tests on theta power for each visibility level showed that the difference between semantic and response conflict was not significant for high (t_21_ = 1.03, p = 0.314) or low (t < 1) visible trials. Therefore we cannot conclude that mid-frontal theta was increased *more* during response conflict than during semantic conflict in both visibility conditions^46^.

Similarly, an ANOVA on parietal alpha-band power yielded main effects of Visibility (F_1,21_ = 7.65, p = 0.012) and Congruency (F_2,42_ = 36.85, p < 0.001), and a significant two-way interaction (F_2,42_ = 16.28, p < 0.001) between Visibility and Congruency. Further analyses indicated that the high visible trials (M = −1.47 dB, SE = 0.31) evoked less alpha power than the low visible trials (M = −1.63 dB, SE = 0.33). Only semantic conflict (M = −0.08 dB, SE = 0.03, t_21_ = −2.27, p = 0.034), and not response conflict (M = −0.05 dB, SE = 0.05, t_21_ = −1.26, p = 0.223), was significant in the low visible trials. Instead, both semantic (M = −0.36 dB, SE = 0.06, t_21_ = −5.41, p < 0.001) and response conflict (M = −0.31 dB, SE = 0.08, t_21_ = −3.64, p = 0.002) were significant in the high visible trials (Fig. 2D). When we separately compared semantic and response conflict for each visibility level, the results showed that the difference between them was not significant for both high and low visible trials (ts < 1). Thus, we cannot conclude that parietal alpha decreased *stronger* during semantic conflict than during response conflict in both visibility conditions^46^.

### Correlational analyses between single trial RT and its frequency-band specific power

In line with previous observations^45^, the results of the cluster-based correlational analyses (see methods) across conditions showed that mid-frontal (Fig. 3A,B) theta power correlated positively with RTs, indicating that trials with longer RTs have more theta power around 400–600 ms after stimulus presentation. In contrast, at the parietal ROI (Fig. 3C,D) alpha power was negatively correlated with RT, indicating that trials with longer RT have less alpha power at that time frequency point. These correlational effects were very general and did not differ between conflict conditions (all ts < 1) in both low and high visible trials. These results are nicely in line with previous findings in conflict tasks showing that frontal theta-band dynamics are tightly coupled to response time^47^, but also extend these results towards parietal alpha, which is to our knowledge, a new observation. Furthermore, there was no significant (t_21_ = −1.36, p = 0.19) difference across all conditions between the mid-frontal ROI and the parietal ROI.

### Discrimination results

The results of 2AFC discrimination task showed that d’ was higher than zero (above chance) in both low (d’ = 0.39, t_21_ = 4.80, p < 0.001, corresponding to 56.1% correct) and high (d’ = 2.51, t_21_ = 12.94, p < 0.001, corresponding to 87.5% correct) visible trials, and the difference between them was significant (M = 2.12, t_21_ = 11.66, p < 0.001). Thus, crucial for the current study, our masking manipulation clearly created two “awareness conditions”: a low visibility and a high visibility condition allowing us to investigate the influence of conflict awareness on the neural processes of semantic and response conflict. However, to be clear, on a group level, the low visible trials cannot be considered to be unconscious.

## Discussion

In the current study we used a masked Stroop priming task to explore how semantic and response conflict at different levels of conflict awareness are processed in the human brain. To dissociate semantic and response conflict in the same task, we used four color words and four colored patches with a four-to-two mapping between the target patch and the required response. Therefore, three types of trials could be defined: congruent (CO), response incongruent (RI), and stimulus incongruent trials (SI). The SI-CO contrast taps into semantic conflict only and the RI-SI purely isolates response conflict^9^,^29^,^34^.

In behavior, we observed response conflict effects both in high and low visible trials. In contrast, semantic conflict was only present in high visible trials. Therefore, based on behavior only, one would conclude that semantic analysis of the color word did not contribute to the overall conflict effect. However, the EEG data revealed a different, more nuanced, picture. Mid-frontal theta power was enhanced during response conflict in both high and low visible trials, but, in theta, semantic conflict was only significant for high visible trials, in accordance with the behavioral results. In contrast, parietal alpha-band power was enhanced during the processing of semantic conflict, both for low and high visible trials. Response conflict in alpha only reached significance in high visible trials. However, there were no significant differences between semantic conflict and response conflict for mid-frontal theta as well as parietal alpha, for both high and low-visible trials. Therefore, we cannot conclude that, for example, mid-frontal theta was increased *more* for response conflict than for semantic conflict in the low-visible condition. Note that we therefore cannot firmly conclude that semantic conflict is specific for parietal alpha-band and response conflict to mid-frontal theta-band power modulations in the low visible condition (this was clearly not the case in the high visible condition)^46^. In general, these EEG findings show that both semantic conflict and response conflict are both processed at different levels of awareness.

Theta-band related conflict dynamics are often observed in conflict tasks such as the Flanker and the Stroop task^42^ and researchers have revealed that this may reflect conflict detection in the medial frontal cortex^40^,^43^,^48^,^49^. Our findings are consistent with Nigbur and colleagues^43^, who also found that stimulus conflict and response conflict elicited an increase of neural oscillations in the theta-band at mid-frontal electrode sites. Our results also showed that these theta oscillations may also reflect semantic conflict that results from the semantic incongruency between the semantic meaning of the prime and the semantic meaning of the target, especially in high visible trials. Our results are also consistent with previous ERP studies, which showed that both stimulus- and response-related conflict evoke an enhancement of the mid-frontal N2^29^ and mid-frontal N450 ERP components^30^. Thus, mid-frontal theta may be a reflection of a general conflict detection and monitoring mechanism of the medial frontal cortex^34^,^41^,^42^,^43^,^50^.

The decrease in alpha power (also referred to as alpha desynchronization) at the parietal region of interest has long been linked to an inhibitory mechanism that gates and filters sensory information^51^,^52^. This inhibitory account fits well with our current results. As shown in Fig. 2C,D, parietal alpha power was reduced in incongruent compared to congruent trials, which is consistent with typical findings in cognitive control studies^53^,^54^. For example, a recent study using the Stroop task found that parietal alpha power was reduced in conflict trials relative to no-conflict, as well as after errors^54^. Alpha power decrease may reflect the refocusing of attention or the increase in alertness after conflict or errors to amplify task-relevant and/or inhibit task-irrelevant stimulus features, and thereby minimizing subsequent conflict and optimizing behavioral adjustments and future decision making^43^,^52^,^54^,^55^,^56^. These behavioral adjustments might be implemented in a top-down manner by a general frontal-parietal cognitive (attention) control network, in which the (posterior) parietal cortex (PPC) plays a key role^34^,^41^,^43^. Previous studies have reported that the PPC might play an important role in the resolution of stimulus-triggered conflict^57^,^58^ and in motor preparation or visuomotor integration during response execution^59^,^60^. Moreover, it has been suggested that alpha power decreases may reflect access to semantic information^51^, which might also explain parts of the reported results here. Therefore, although the decrease in posterior alpha-band power in both semantic and response conflict fits with well with recent findings, future studies should explore this finding in more detail.

Finally, there has been debate about whether conflict control processing is specific for various origins of conflict (stimulus, response) or whether it is more global (or “domain general”), recruiting a similar network irrespective the source of conflict^33^,^58^. In the present study, response conflict and semantic conflict were both related to mid-frontal theta-band and parietal alpha-band modulations (especially clear in the high visible trials), suggesting that the oscillatory processes related to both kinds of conflict are not completely independent^34^. Some previous studies (using fully conscious stimuli only) have suggested that conflict processing might be domain-specific^8^,^10^,^27^,^28^, but overall results from neuroimaging have been inconsistent. On the other hand, several other studies found that conflict control are relatively domain general^29^,^30^ or entails a mix of domain specific and domain general processes^31^,^32^,^33^,^34^,^58^. The inconsistent findings across studies may reflect the flexibility of conflict processing in the human brain, because it seems that the exact manner in which conflict control is implemented strongly depends on the task context. Few studies however have tested the role of awareness in conflict processing, and future studies are needed to further clarify the potential influence of conflict awareness on the (neural) processing of semantic and response conflict in different task contexts and paradigms. This is especially relevant because in this study we did not block awareness completely of the prime stimulus (hence referred to as the low visible condition). Future studies, using stronger awareness manipulations, are necessary to explore the neural oscillatory mechanisms involved during the processing of fully unconscious semantic and response conflict.

## Additional Information

**How to cite this article**: Jiang, J. *et al.* EEG neural oscillatory dynamics reveal semantic and response conflict at difference levels of conflict awareness. *Sci. Rep.* **5**, 12008; doi: 10.1038/srep12008 (2015).

## Supplementary Material

Supplementary Information
