# Supplementary material for: EEG neural oscillatory dynamics reveal semantic and response conflict at difference levels of conflict awareness
Source: Sci Rep. 2015 Jul 14;5:12008. doi: 10.1038/srep12008 (PMC4500944; doi:10.1038/srep12008)
Supplement: Supplementary Information [file srep12008-s1.doc]

**Supplementary material:**

**EEG neural oscillatory dynamics reveal semantic and response conflict at difference levels of conflict awareness**

Jun Jiang, Qinglin Zhang, Simon van Gaal


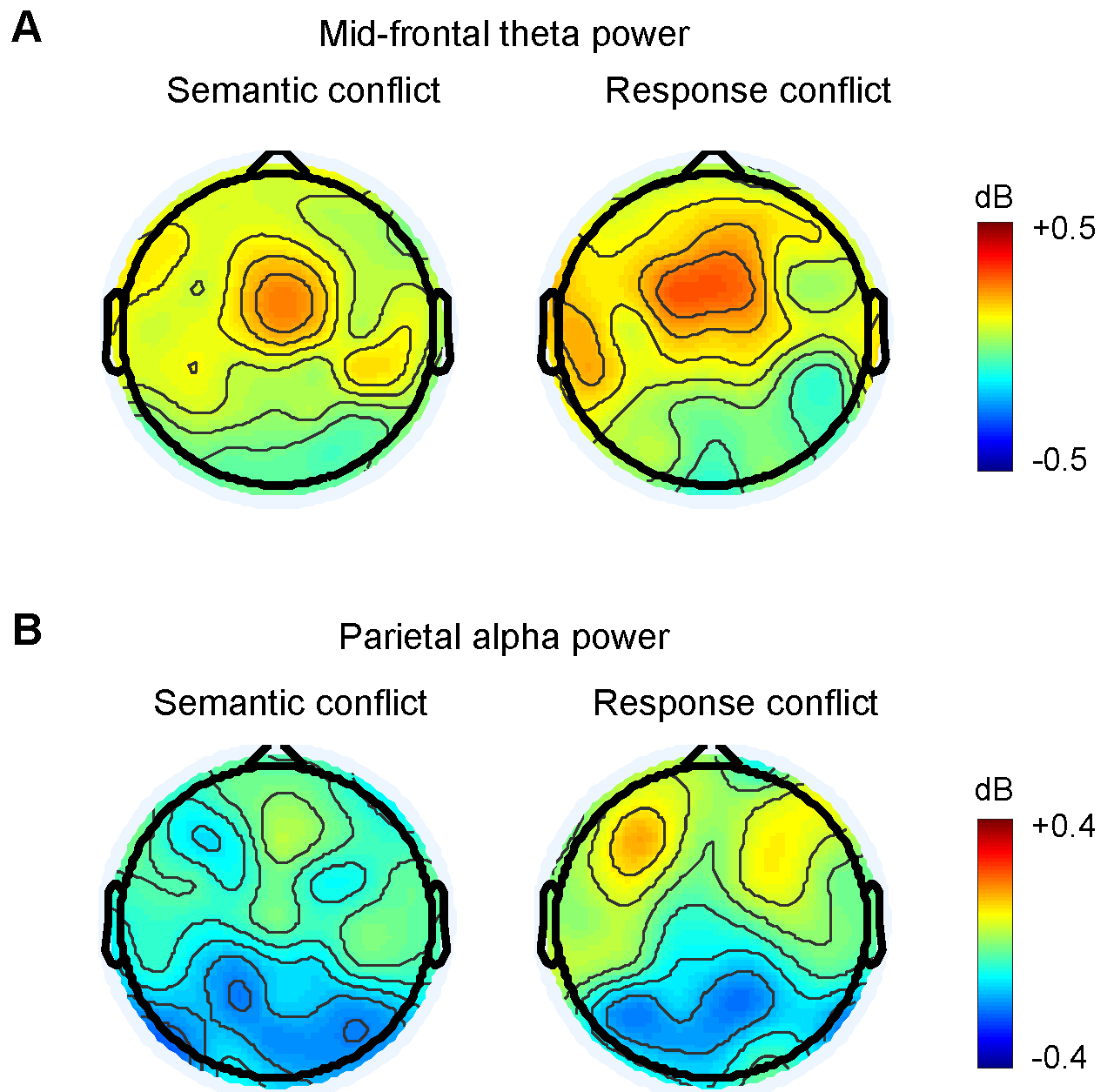


**Figure S1**. Topographical plots for semantic conflict and response conflict. **(A)** Topographical plots for semantic conflict (semantic incongruent-congruent) and response conflict (response incongruent-semantic incongruent) across visibility conditions for theta-band power. The T-F ROI is defined based on the results reported in Figure 2A. The plots show that the topographical distribution of the theta power increase due to semantic conflict and response conflict is similar and centered at mid-frontal electrode sites. **(B)** Topographical plots for semantic conflict and response conflict across visibility conditions for alpha-band power. The T-F ROI is defined based on the results reported in Figure 2B. The plots show that the topographical distribution of alpha power increases due to semantic conflict and response conflict are similar and centered at parietal electrode sites.


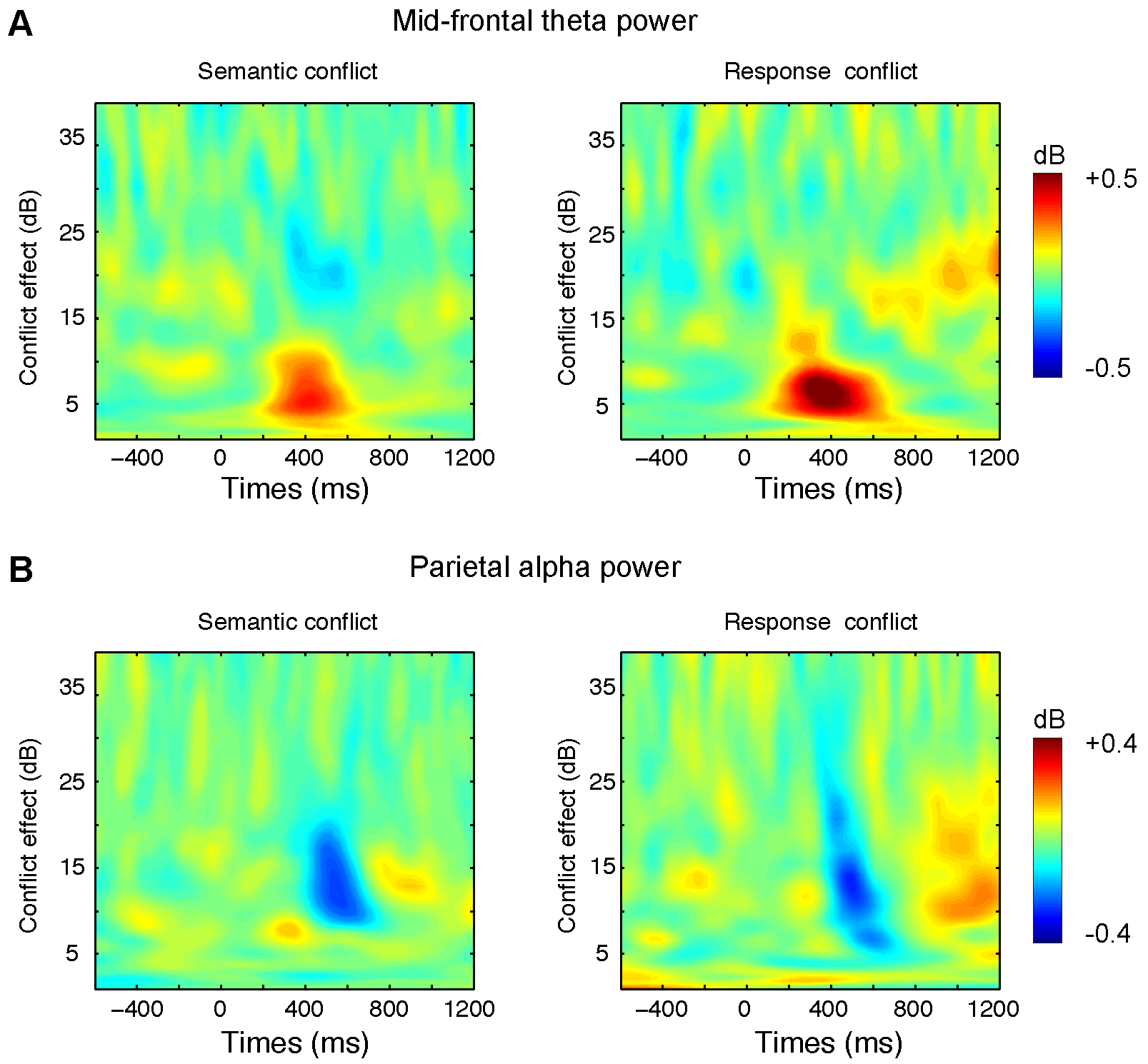


**Figure S2.** Time-frequency plots for semantic conflict and response conflict. **(A)** Time-frequency plots for semantic conflict (semantic incongruent-congruent) and response conflict (response incongruent-semantic incongruent) across visibility conditions at the mid-frontal ROI. The plots show that the power distribution of semantic and response conflict at the mid-frontal ROI is similar. **(B)** Time-frequency plots for semantic conflict and response conflict across visibility conditions at the parietal ROI. The plots show that the power distribution of semantic and response conflict at parietal ROI is similar. Time 0 is the target onset in all panels.
